# *EYA4* gene functions as a prognostic marker and inhibits the growth of intrahepatic cholangiocarcinoma

**DOI:** 10.1186/s40880-016-0133-z

**Published:** 2016-07-28

**Authors:** Xiao-Yi Hao, Jian-Peng Cai, Xin Liu, Wei Chen, Xun Hou, Dong Chen, Jia-ming Lai, Li-Jian Liang, Xiao-Yu Yin

**Affiliations:** Departments of Pancreatobiliary Surgery, The First Affiliated Hospital, Sun Yat-sen University, Zhongshan 2nd Road, Guangzhou, 510080 Guangdong P. R. China

**Keywords:** *EYA4* gene, Intrahepatic cholangiocarcinoma, Prognostic factor, Surgical resection, Tumor suppressor gene

## Abstract

**Background:**

The molecular prognostic markers and carcinogenesis of intrahepatic cholangiocarcinoma (ICC) have not been well documented. The purpose of this study was to investigate the prognostic value of the eyes absent homolog 4 (*EYA4*) gene in ICC and its biological effects on ICC growth in vitro and in vivo.

**Methods:**

One hundred twelve patients with ICC who underwent hepatectomy were enrolled in the study. *EYA4* mRNA and EYA4 protein levels in ICC and adjacent non-tumoral tissues were evaluated using real-time quantitative polymerase chain reaction and immunohistochemical staining, respectively. EYA4 protein levels in ICC cells were determined using western blot analysis. The associations between EYA4 expression and clinicopathologic features of ICC were analyzed. To identify independent prognostic factors, univariate and multivariate analyses were performed. The biological effects of *EYA4* on ICC cells were evaluated by establishing stable *EYA4*-overexpressing transfectants in vitro, and *EYA4*’s effects on tumor growth were evaluated by intra-tumoral injection of *EYA4*-expressing plasmids in a NOD/SCID murine model of xenograft tumors.

**Results:**

ICC tissues had significantly lower *EYA4* mRNA and protein levels compared with adjacent non-tumoral tissues (both *P* < 0.001). Univariate and multivariate analyses showed that EYA4 protein level, tumor number, adjacent organ invasion, lymph node metastasis, and tumor differentiation were independent prognostic factors for disease-free survival and overall survival (all *P* < 0.05). In vitro, *EYA4* overexpression inhibited tumor cell growth, foci formation, and cell invasiveness. In vivo, intra-tumoral injection of *EYA4*-expressing plasmids significantly inhibited ICC growth in the murine xenograft model compared with the control group (*P* < 0.05).

**Conclusion:**

*EYA4* gene functioned as a molecular prognostic marker in ICC, and its overexpression inhibited tumor growth in vitro and in vivo.

## Background

Over the past four decades, intrahepatic cholangiocarcinoma (ICC) has had a rising incidence worldwide [1, 2]. Surgical resection offers a chance for cure for patients with ICC, however its long-term outcome is still dismal due to a high incidence of postoperative tumor recurrence and metastases. The 5-year overall survival (OS) rate of ICC patients has been found to be only 15.0%–30.7% [3–5]. Although some clinicopathologic parameters, including serum carbohydrate antigen 19-9 (CA19-9) level, tumor number, adjacent organ invasion, lymph node metastasis, and tumor differentiation, have been shown to be prognostic factors for ICC [5–7], the molecular prognostic markers and potential mechanisms of ICC have not been well documented. Elucidating the molecular prognostic markers of ICC would be clinically and scientifically significant, since it would help researchers understand the carcinogenesis of ICC and offer potential therapeutic targets.

Eyes absent homolog 4 (*EYA4*) is a member of the *EYA* gene family, which, in mammals, contains four members: *EYA1*, *EYA2*, *EYA3*, and *EYA4*. The *EYA* gene family was first discovered in drosophila eye development, in which its mutation or deletion led to the “eyeless” phenotype, and its mis-expression led to the formation of ectopic eye tissue [8, 9]. Some studies showed that *EYA* family members could combine with sine oculis (*SIX1*) and dachshund (*DACH*) to function as transcriptional factors to regulate specific gene expression in mammalian organogenesis [10, 11]. In addition, *EYA* family members had dual functions of threonine and tyrosine phosphatases [11–14]. However, its implications in carcinogenesis are unclear.

Using DNA methylation microarray, our previous study showed that the *EYA4* gene was markedly hypermethylated in hepatocellular carcinoma (HCC) tissues compared with adjacent non-tumorous tissues [15]. Low *EYA4* expression in HCC tissues was associated with short disease-free survival (DFS) and OS, and multivariate analysis showed that *EYA4* expression was an independent prognostic factor in HCC patients [15]. These results suggested that the *EYA4* gene might play an important role in tumor occurrence and progression, but its prognostic value in ICC and its biological effects on ICC cells remain unknown.

The purpose of this study was to assess, by univariate and multivariate survival analyses, the prognostic value of the *EYA4* gene in ICC and to investigate its biological effects on ICC cells in vitro and in vivo.

## Methods

### Patients and specimens

One hundred and twelve patients with histologically proven ICC, who underwent curative hepatectomy at the First Affiliated Hospital of Sun Yat-sen University, in Guangzhou, Guangdong, China between June 2006 and June 2012, were included in this study. The inclusion criteria of the study were as follows: (1) histologically diagnosed ICC; (2) curative resection of tumors; and (3) absence of distant metastases. The patients who met one of the following criteria were excluded from the study: (1) perihilar cholangiocarcinoma (Klatskin tumor); (2) mix tumors of HCC and ICC; (3) R1 or R2 resection or laparotomy with tumor biopsy; and (4) receiving neoadjuvant chemotherapy and/or radiotherapy.

Of the 112 patients included in this study, 63 (56.2%) were men, and 49 (43.8%) were women, with a median age of 57 years (range, 28–79 years). Eighty-six patients (76.8%) had a single tumor; the remaining 26 patients (23.2%) had multiple tumors. Tumor size ranged from 1.0 to 16.0 cm in diameter (median, 5.7 cm). Fourteen patients (12.5%) had adjacent organ invasion. Forty-nine patients (43.8%) had lymph node metastasis. Seventy patients (62.5%) had an elevated level of serum CA19-9 (>37 U/L), and 43 (38.4%) had an elevated serum level of carcino-embryonic antigen (CEA) (>5 µg/L). All patients underwent curative hepatectomy with regional lymph node dissection. According to the American Joint Committee on Cancer (AJCC) *Cancer Staging Manual* (7th edition) [16], of the 112 ICC patients, 43 (38.4%) had tumor-node-metastasis (TNM) stage I disease, 10 (8.9%) had TNM stage II disease, 6 (5.4%) had TNM stage III disease, and 53 (47.3%) had TMN stage IV disease.

Patients were followed up every 1–3 months, ending in March 2014. Tumor recurrence/metastasis was diagnosed on the basis of dynamic imaging results (i.e., contrast-enhanced computed tomography and/or contrast-enhanced magnetic resonance imaging and/or contrast-enhanced ultrasonography), and serum CA19-9 and CEA levels.

For 48 patients, tumorous and adjacent non-tumorous tissues were collected, snap-frozen instantly in liquid nitrogen, and stored at −80 °C for molecular biological analysis. Paraffin-embedded ICC specimens of 112 patients that were used for immunohistochemical staining were obtained from the Department of Pathology, First Affiliated Hospital of Sun Yat-sen University.

This study was approved by the Ethics Committee of the First Affiliated Hospital of Sun Yat-sen University. Written informed consent was obtained from each patient.

### Real-time quantitative polymerase chain reaction (RT-qPCR)

Using RNAiso (TaKaRa, Dalian, Liaoning, China), total RNA was extracted from 48 pairs of tumorous and adjacent non-tumorous tissues as well as cultured cells. Using the PrimeScript^®^ RT Reagent kit (TaKaRa), reverse transcription was performed with 0.5 μg of total RNA. RT-qPCR was performed to examine the mRNA level of *EYA4* using TaKaRa SYBR^®^ Premix Ex Taq™ Kit (TaKaRa) and the ABI PRISM^®^ 7900HT RT-qPCR System (Applied Biosystems, Carlsbad, CA, USA). Glyceraldehyde-3-phosphate dehydrogenase (*GAPDH*) gene was used as endogenous control. Primers were listed as follows: *EYA4* sense 5′-GAATAACACAGCCGATGG-3′, antisense 5′-CCAGGTCACTATCAGGAG-3′; *GAPDH* sense 5′-GCACCGTCAAGGCTGAGAAC-3′, antisense 5′-TGGTGAAGACGCCAGTGGA-3′. Thermocycling conditions for PCR were as follows: an initial cycle of 95 °C for 5 min, followed by 40 cycles of 95 °C for 30 s, 95 °C for 5 s, and 65 °C for 30 s, and finally 72 °C for 5 min for the extension. ΔCt (difference in cycle threshold) was calculated for each sample (ΔCt = Ct_Target gene_ − Ct_GAPDH_), and relative quantities were compared. All reactions were repeated in triplicate.

### Immunohistochemical (IHC) staining

Paraffin-embedded tissues were cut into 4-μm sections and mounted on glass slides. IHC analysis was performed on 112 ICC tissues to detect EYA4 protein. Briefly, tissues were deparaffinized in dimethylbenzene, rehydrated in graded alcohol, and then incubated in 3% H_2_O_2_ to block endogenous peroxidase activity. Antigen retrieval was achieved by treating the tissues with citrate buffer in a pressure cooker. Tissues were subsequently incubated with rabbit anti-human EYA4 polyclonal antibodies (dilution 1:25; Abcam, Cambridge, UK) at 4 °C overnight. A ChemMate™ Envision™ Detection Kit (Dako, Glostrup, Denmark) was used to detect and visualize the bound primary antibodies. Human skeletal muscle tissue was used as positive control for EYA4 antibody (recommended by supplier), and the rabbit IgG antibody (Biosynthesis, Beijing, China) was used as negative control in IHC staining. Staining intensity (negative, 0; mild, 1; moderate, 2; and severe, 3) and proportion of positive cells (negative, 0; ≤10, 1; >10 and ≤33%, 2; >33 and ≤66%, 3; and >66%, 4) were quantified, respectively [17]. Summation of the scores of the two parameters represents protein expression level. Each slide was scored by two observers independently, and the average of their scores was recorded as the IHC score.

### Cell lines

Human ICC cell lines RBE and SSP-25 were obtained from the Cell Resources Center of Shanghai Institutes for Biological Science, Chinese Academy of Science (Shanghai, China). Cells were cultured in RPMI-1640 (Gibco BRL, Rockville, MD, USA) and supplemented with 10% fetal bovine serum (Gibco BRL).

### Western blotting

Total cell lysates were prepared using a KeyGEN Total Protein Extraction Kit (Nanjing, Jiangsu, China). Aliquots of 10–20 μL cell lysates were electrophoresed in 10% sodium dodecyl sulfate polyacrylamide gel electrophores (SDS-PAGE), and proteins were transferred onto polyvinylidene fluoride (PVDF) membrane (Merck Millipore, Cambridge, UK). The membrane was blocked, incubated with primary antibody at 4 °C overnight, and then incubated with secondary antibody at room temperature for 30 min. Rabbit anti-human EYA4 polyclonal antibodies (dilution 1:300; Abcam), GAPDH monoclonal antibody, and goat anti-rabbit secondary antibody (dilution 1:3000; Biosynthesis) were used to detect EYA4 and GAPDH protein. GAPDH was used as loading control. Bands were visualized using an ECL kit (KeyGEN, Nanjing, China) and exposed to Kodak X-OMAT film (Carestream Health Inc., Rochester, NY, USA).

### Establishment of stable *EYA4*-overexpressing transfectants of ICC cells

The pReceiver-M02 empty vector and the recombinant pReceiver-M02/*EYA4* overexpression plasmids (U0188) were purchased from Genecopoeia (Rockville, MD, USA). The recombinant *EYA4*-expressing plasmid was transfected into RBE and SSP-25 cells at 70%–80% confluence using Lipofectamine™ 2000 (Invitrogen, Carlsbad, CA, USA). Plasmid pReceiver-M02 was used as control (FulenGen, Guangzhou, Guangdong, China). After 2 weeks of G418 selection, stable transfected cells were subjected to limited dilution. Survival clones with the highest expression of *EYA4* in both cell lines were used for further studies. Stable *EYA4*-overexpressing transfectants and vector transfectants of RBE and SSP-25 cells were designated as RBE-EYA4, RBE-Vector, SSP-EYA4, and SSP-Vector, respectively.

### Cell proliferation assay

Cells were seeded into 96-well plates at the density of 2000 cells per well. Cell proliferation was detected at 24, 48, 72, and 96 h after seeding using Cell Counting Kit-8 (Dojindo, Kumamoto, Japan). Absorbance values at 450 nm (A_450_) were recorded as representation of cell viability. Each experiment was done in triplicate, and four individual experiments were performed.

### Foci formation assay

Cells were seeded into 6-well plates at the density of 1000 cells per well and cultured for 7 days. After fixing in 4% paraformaldehyde and staining with 1% crystal violet, foci with more than 50 cells were counted. Four independent experiments were performed.

### Cell invasion assay

Cell invasion assay was performed with BD BioCoat™ Tumor Invasion System (BD Biosciences, Bedford, MA, USA). Approximately 5 × 10^4^ cells/chamber were seeded into the rehydrated chamber. After 22 h of incubation, cells that invaded to the bottom surface of the chamber were fixed and stained as mentioned above, then counted under the microscope. Four assays were performed.

### In vivo experiments using xenograft ICC in NOD/SCID mice

The cultured RBE and SSP-25 cells were trypsinized and resuspended to a density of 1 × 10^8^ cells/mL. Then, 1 × 10^7^ RBE or SSP-25 cells in 100 µL were inoculated subcutaneously into both flanks of 4–5-week male NOD/SCID mice (HFK Bioscience Co. Ltd, Beijing, China). After the xenograft tumors reached 6 mm in diameter (or volume >100 mm^3^), the RBE and SSP-25 tumor-bearing mice were randomized into EYA4, vector, and blank groups (in each group, there were three mice bearing 5 tumors) and subjected to the following treatments, respectively. (1) EYA4 group: intra-tumoral injection of lipofactamine 2000, *EYA4*-expressing plasmids, and Opti-MEM; (2) Vector group: intra-tumoral injection of lipofactamine 2000, vector-plasmids, and Opti-MEM; and (3) Blank group: intra-tumoral injection of lipofectamine 2000 and Opti-MEM. Each injection contained 20 μg plasmid (1 μg/μL) mixed with 40 μL lipofectamine 2000 and 20 μL Opti-MEM in EYA4 and vector groups; in blank group, 40 μL lipofectamine 2000 mixed with 2 μL Opti-MEM was injected each time. The treatment was repeated every 5 days and given a total of 6 times. Body weights of mice and the tumor size were monitored every 3 days. Tumor volume was calculated as follows: volume (mm^3^) = length × width^2^/2. On post-treatment day 30, the mice were euthanized, and their tumors were removed and weighed. The in vivo experiments were approved by the Ethics Committee of the First Affiliated Hospital of Sun Yat-sen University.

### Statistical analysis

Results were presented as mean ± standard deviation (SD) or median (range). Statistical analysis was performed using SPSS 17.0 software (IBM, Chicago, IL, USA). DFS was calculated from the date of surgery to the date that tumor recurrences or metastases were confirmed. OS was defined as the interval between the date of surgery and death or the date of last follow-up. DFS and OS were calculated using the Kaplan–Meier method. The associations between EYA4 protein expression and clinicopathologic parameters were analyzed using the Mann–Whitney *U* test. Univariate and multivariate Cox regression analyses were used to identify independent prognostic factors. Inter-group comparisons of foci formation, cell invasion ability, mice weight, and xenograft tumor size and weight were done using Student’s *t* test. Cell proliferation and the xenograft tumor growth curve were compared among groups by repeated measures of analysis of variance. Two-sided *P* < 0.05 values were considered statistically significant.

## Results

### Postoperative tumor recurrence and survival

Of the 112 patients, 107 (95.5%) had a regular follow-up, with a median follow-up of 13.9 months (range, 2.5–71.7 months). The remaining 5 patients defaulted after a follow-up of 8.0, 13.0, 15.0, 18.3, and 24.0 months, respectively. Tumor recurrence developed in 83 patients, with a median time to recurrence of 5.5 months (range, 1.0–32.0 months). Seventy-six patients died during follow-up, with a median survival of 11.5 months (range, 2.5–42.0 months). The 1-, 3-, and 5-year DFS rates were 47.7%, 22.0% and 22.0%, respectively; the 1-, 3-, and 5-year OS rates were 64.3%, 30.5% and 23.1%, respectively.

### *EYA4* expression in ICC tissues and its clinical values

**Fig. 1:**
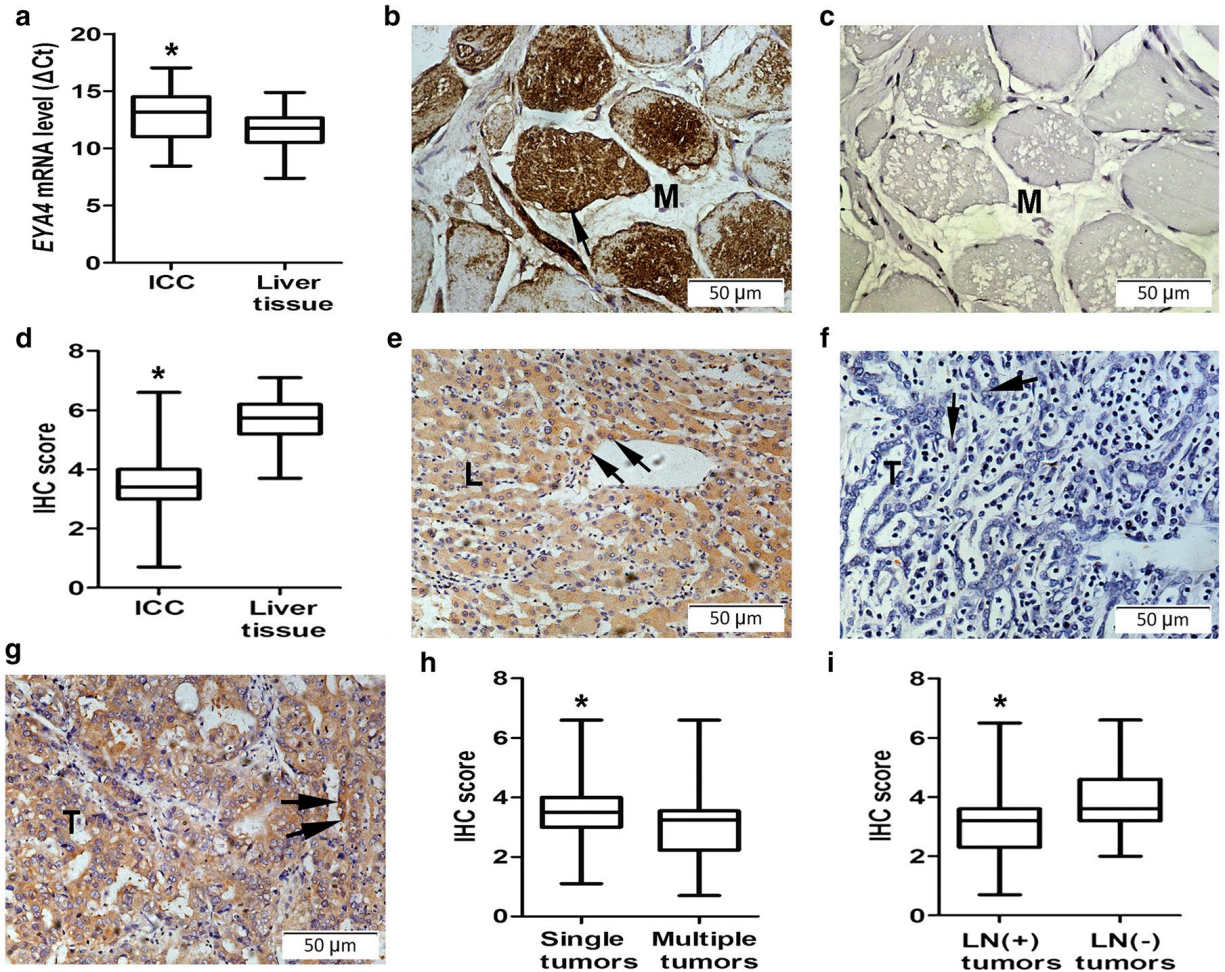
Eyes absent homolog 4 (*EYA4)* mRNA and protein expression in intrahepatic cholangiocarcinoma (ICC) tissues. **a** Boxplot of *EYA4* real-time quantitative polymerase chain reaction shows that ICC tissues have lower *EYA4* mRNA levels than non-tumorous adjacent liver tissues (*P < 0.001). ΔCt stands for difference in cycle threshold; it was calculated as follows: ΔCt = Ct_Target gene_ − Ct_GAPDH_. **b** Positive control staining of human skeleton muscle tissue using EYA4 antibody. **c** Negative control staining of human skeleton muscle using rabbit IgG as the primary antibody. **d** Boxplot of EYA4 immunohistochemical (IHC) scores shows that ICC tissues have lower EYA4 protein level than non-tumorous adjacent liver tissues (*P < 0.001). **e** A typical staining of adjacent non-tumorous liver tissue. **f** A weak staining in tumor tissues with lymph node metastasis. **g** A strong staining in tumor tissues without lymph node metastasis. *Black arrows* indicate positive staining of hepatocytes and ICC cells. *L* liver. *T* tumor. *M* muscle. **h** Boxplot of EYA4 IHC scores shows that multiple tumors have lower EYA4 protein levels than single tumors (*P = 0.023). **i** Boxplot of EYA4 IHC scores shows that tumor with lymph node metastasis have lower EYA4 protein levels than those without lymph node metastasis (*P < 0.001)

*EYA4* mRNA level in ICC tissues was significantly lower than that in adjacent non-tumorous tissues, with a median ΔCt of 13.14 (range, 8.45–17.03) vs 11.77 (range, 7.38–14.87; *P* < 0.001; Fig. 1a). Consistently, EYA4 protein level in ICC tissues was significantly lower than that in non-tumorous tissues, with a median IHC score of 3.4 (range, 0.7–6.6) vs 5.7 (range, 3.7–7.1; *P* < 0.001; Fig. 1b–g). With respect to associations between EYA4 expression and clinicopathologic features, multiple tumors had a lower EYA4 protein level than single tumors, with a median IHC score of 3.2 (range, 0.7–6.6) vs 3.5 (range, 1.1–6.6; *P* = 0.023; Fig. 1h). Furthermore, tumors with lymph node metastases had a lower EYA4 protein level than tumors without lymph node metastases, with a median IHC score of 3.2 (range, 0.7–6.5) vs 3.6 (range, 2.0–6.6; *P* < 0.001; Fig. 1i).

**Fig. 2:**
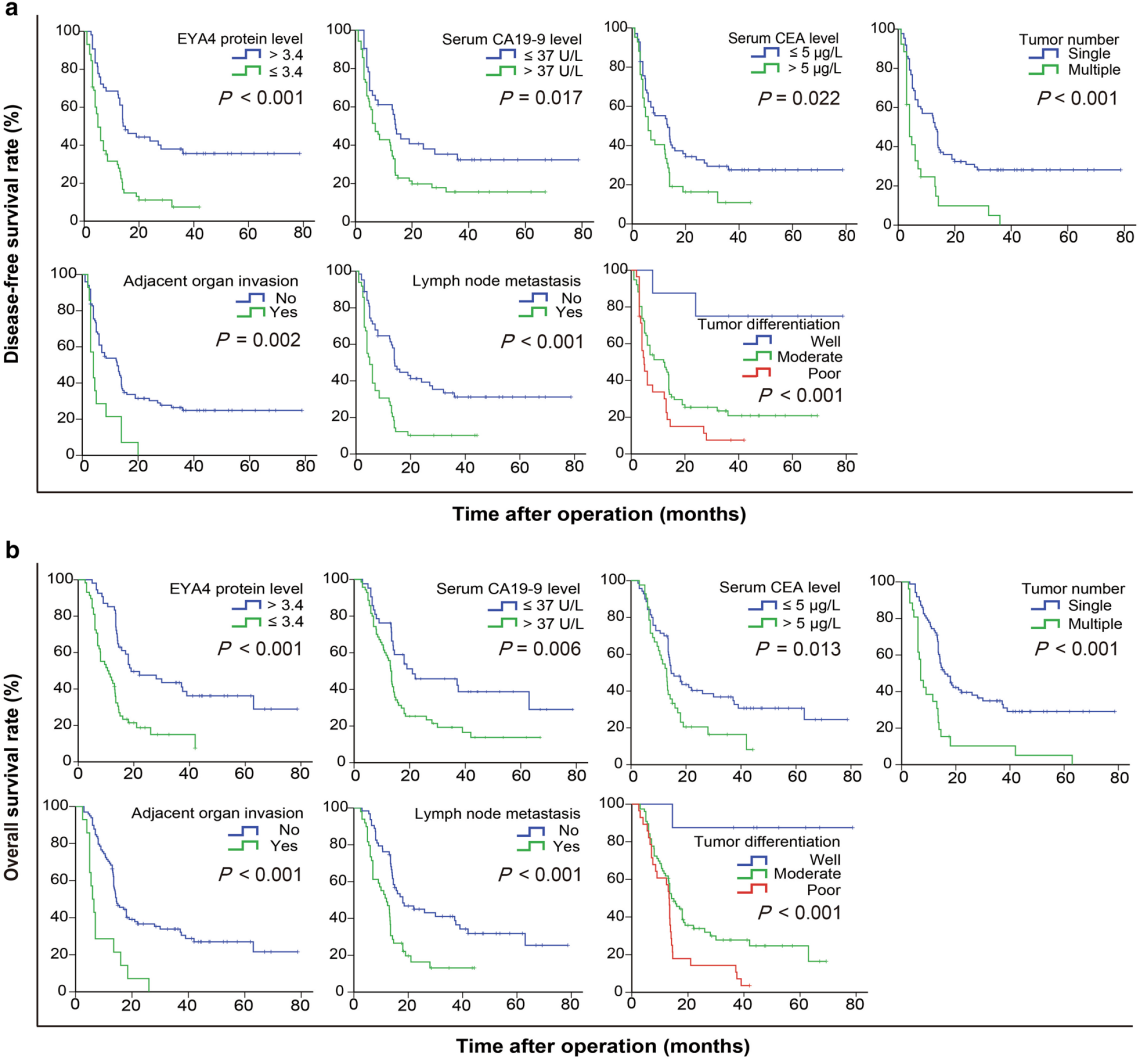
Disease-free survival (DFS) and overall survival (OS) curves of 112 ICC patients. **a** DFS curves of the 112 ICC patients show that patients with lower EYA4 protein levels, serum carbohydrate antigen 19-9 (CA19-9) level >37 U/L, serum CEA level >5 µg/L, multiple tumors, adjacent organ invasion, lymph node metastasis, and poor differentiation had worse DFS (all *P* < 0.05). **b** OS curves of the 112 ICC patients show that patients with lower EYA4 protein levels, serum CA19-9 level >37 U/L, serum CEA level >5 µg/L, multiple tumors, adjacent organ invasion, lymph node metastasis, and poor differentiation had worse OS (all *P* < 0.05)

Univariate analysis showed that EYA4 protein level, serum CA19-9 level, serum CEA level, tumor number, adjacent organ invasion, lymph node metastasis, and tumor differentiation were prognostic factors for DFS and OS (Fig. 2). Multivariate analysis showed that EYA4 protein level, tumor number, adjacent organ invasion, lymph node metastasis, and tumor tumor differentiation were independent prognostic factors for DFS and OS (Table 1). These results suggest that the *EYA4* gene might play an important role in ICC progression.

**Table 1:** Prognostic factors for DFS and OS of patients with intrahepatic cholangiocarcinoma determined by using univariate and multivariate Cox regression models

Variable	DFS			OS		
	HR	95% CI	*P* value	HR	95% CI	*P* value
*Univariate*						
Sex (women vs. men)	0.820	0.532–1.265	0.370	0.853	0.549–1.324	0.478
Age (<65 years vs. ≥65 years)	1.088	0.659–1.797	0.741	0.984	0.583–1.661	0.953
Liver cirrhosis (yes vs. no)	1.410	0.706–2.816	0.331	1.055	0.508–2.191	0.885
EYA4 protein expression^a^ (≤3.4 vs. >3.4)	2.635	1.684–4.122	<0.001	2.730	1.727–4.314	<0.001
CA19-9 (>37 U/L vs. ≤37 U/L)	1.753	1.107–2.778	0.017	1.955	1.214–3.147	0.006
CEA (>5 µg/L vs. ≤5 µg/L)	1.665	1.077–2.574	0.022	1.764	1.128–2.758	0.013
Size of tumor (≤5 cm vs. >5 cm)	0.983	0.631–1.529	0.938	1.047	0.667–1.643	0.841
Number of tumors (multiple vs. single)	2.469	1.521–4.006	<0.001	2.921	1.809–4.717	<0.001
Vascular invasion (yes vs. no)	1.803	0.899–3.616	0.097	2.049	1.053–3.989	0.055
Adjacent organ invasion (yes vs. no)	2.489	1.388–4.462	0.002	3.328	1.851–5.984	<0.001
Lymph node metastasis (yes vs. no)	2.410	1.558–3.728	<0.001	2.279	1.460–3.558	<0.001
Tumor differentiation (poor vs. moderate vs. well)	2.009	1.366–2.953	<0.001	2.272	1.540–3.353	<0.001
*Multivariate*						
EYA4 protein expression^a^ (≤3.4 vs. >3.4)	1.730	1.060–2.823	0.028	1.781	1.093–2.902	0.021
Number of tumors (multiple vs. single)	2.008	1.201–3.357	0.008	2.494	1.508–4.124	<0.001
Adjacent organ invasion (yes vs. no)	2.325	1.238–4.367	0.009	3.765	1.954–7.253	0.001
Lymph node metastasis (yes vs. no)	1.919	1.181–3.120	0.009	1.798	1.106–2.921	0.018
Tumor differentiation (poor vs. moderate vs. well)	2.053	1.301–3.238	0.002	2.530	1.578–4.055	<0.001

*EYA4* Eyes absent homolog 4; *CA19*-*9* carbohydrate antigen 19-9; *CEA* carcino-embryonic antigen; *DFS* disease-free survival; *OS* overall survival; *HR* hazard ratio; *CI* confidence interval

^a^Immunohistochemical (IHC) score, split at median

### *EYA4* overexpression suppressed growth of ICC cells in vitro

**Fig. 3:**
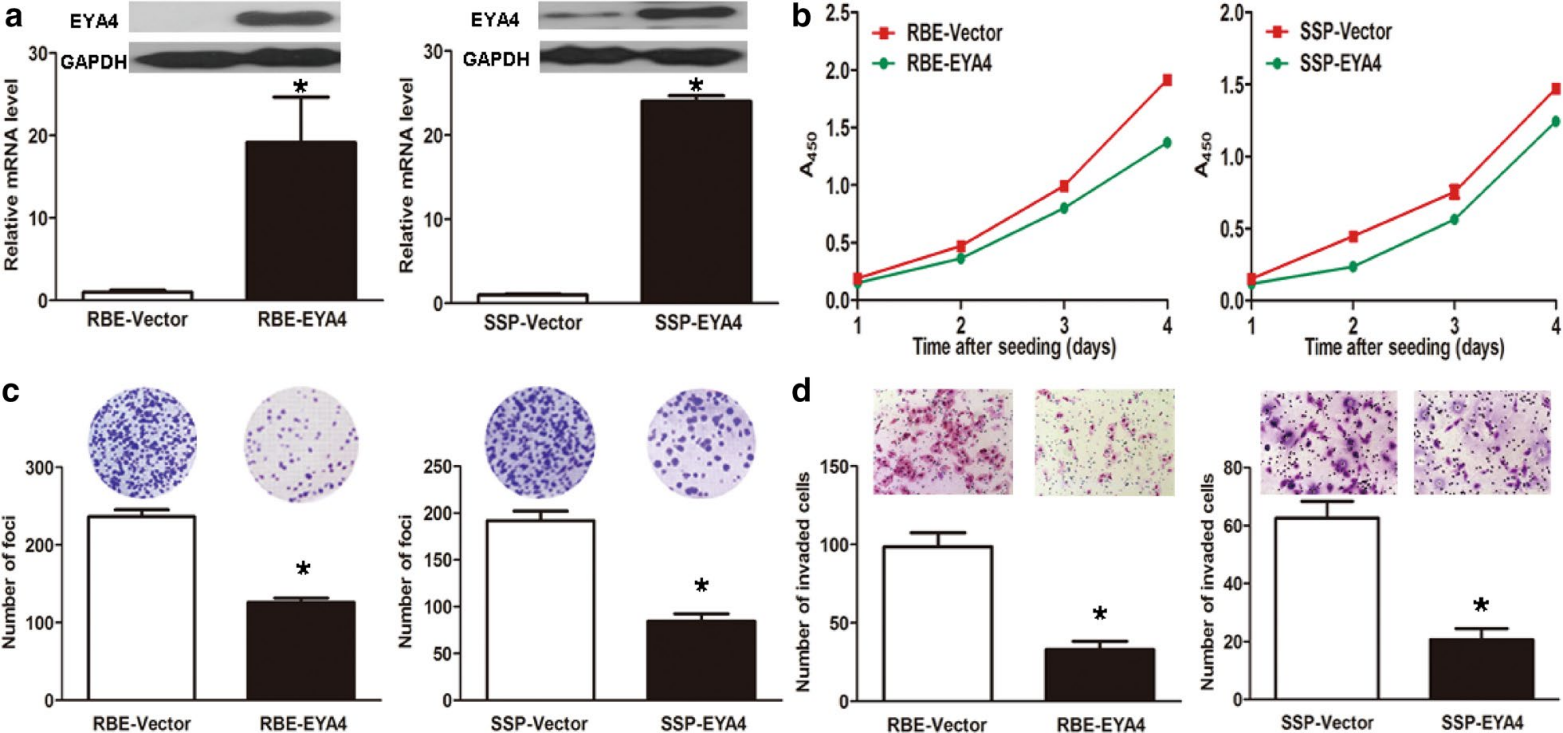
Tumor-suppressive effects of *EYA4* gene expression in vitro. **a** *EYA4*-overexpressing transfectants RBE-EYA4 and SSP-EYA4 show significantly elevated expression levels of *EYA4* mRNA (*P = 0.001 and *P < 0.001, respectively) as well as protein levels. **b** Overexpression of the *EYA4* gene significantly suppressed the growth rate of RBE-EYA4 and SSP-EYA4 compared with RBE-vector and SSP-vector, respectively (both *P = 0.001). **c** *EYA4*-overexpressing transfectants show weaker capacity in foci formation than their vector control transfectants (both *P < 0.001). **d** *EYA4* overexpression resulted in weakened invasiveness of transfectants (both *P < 0.001)

Using two stable *EYA4*-overexpressing transfectants of ICC cell lines RBE-EYA4 and SSP-EYA4, we studied the biological effects of the *EYA4* gene on ICC cells. RT-qPCR showed that *EYA4* mRNA levels in RBE-EYA4 and SSP-EYA4 cells were significantly up-regulated compared with that of their vector control transfectants (19.14 ± 5.51-fold for RBE-EYA4, 24.04 ± 0.65-fold for SSP-EYA4; both *P* < 0.001; Fig. 3a). Consistently, Western blot analysis showed that EYA4 protein levels in RBE-EYA4 and SSP-EYA4 were much higher than those in RBE-vector and SSP-vector, respectively (Fig. 3a).

Using cell proliferation assays, foci formation assays, and cell invasion assays, we evaluated the biological effects of the *EYA4* gene on ICC cells. Cell proliferation assays showed that overexpression of *EYA4* significantly inhibited the growth rate of ICC cells (*P* = 0.001 for both cell lines; Fig. 3b). The frequencies of foci formation in RBE-EYA4 and SSP-EYA4 were significantly lower than those in RBE-Vector and SSP-Vector, with a foci number of 126.0 ± 5.6 vs 236.3 ± 9.1 for RBE-EYA4, and a foci number of 84.3 ± 8.0 vs 192.0 ± 10.2 for SSP-EYA4 (both *P* < 0.001; Fig. 3c). Cell invasion assays showed that the number of invaded RBE-EYA4 and SSP-EYA4 cells were significantly lower than that of invaded RBE-Vector and SSP-Vector cells, with a cell number of 33.0 ± 5.3 vs 98.6 ± 9.0 for REB-EYA4, and a cell number of 20.6 ± 3.8 vs 62.6 ± 5.8 for SSP-EYA4 (both *P* < 0.001; Fig. 3d). These data suggest that overexpression of *EYA4* had considerable tumor-suppressive effects on ICC cells.

### Intra-tumoral injection of *EYA4*-expressing plasmids inhibited the growth of xenograft ICC in NOD/SCID mice

**Fig. 4:**
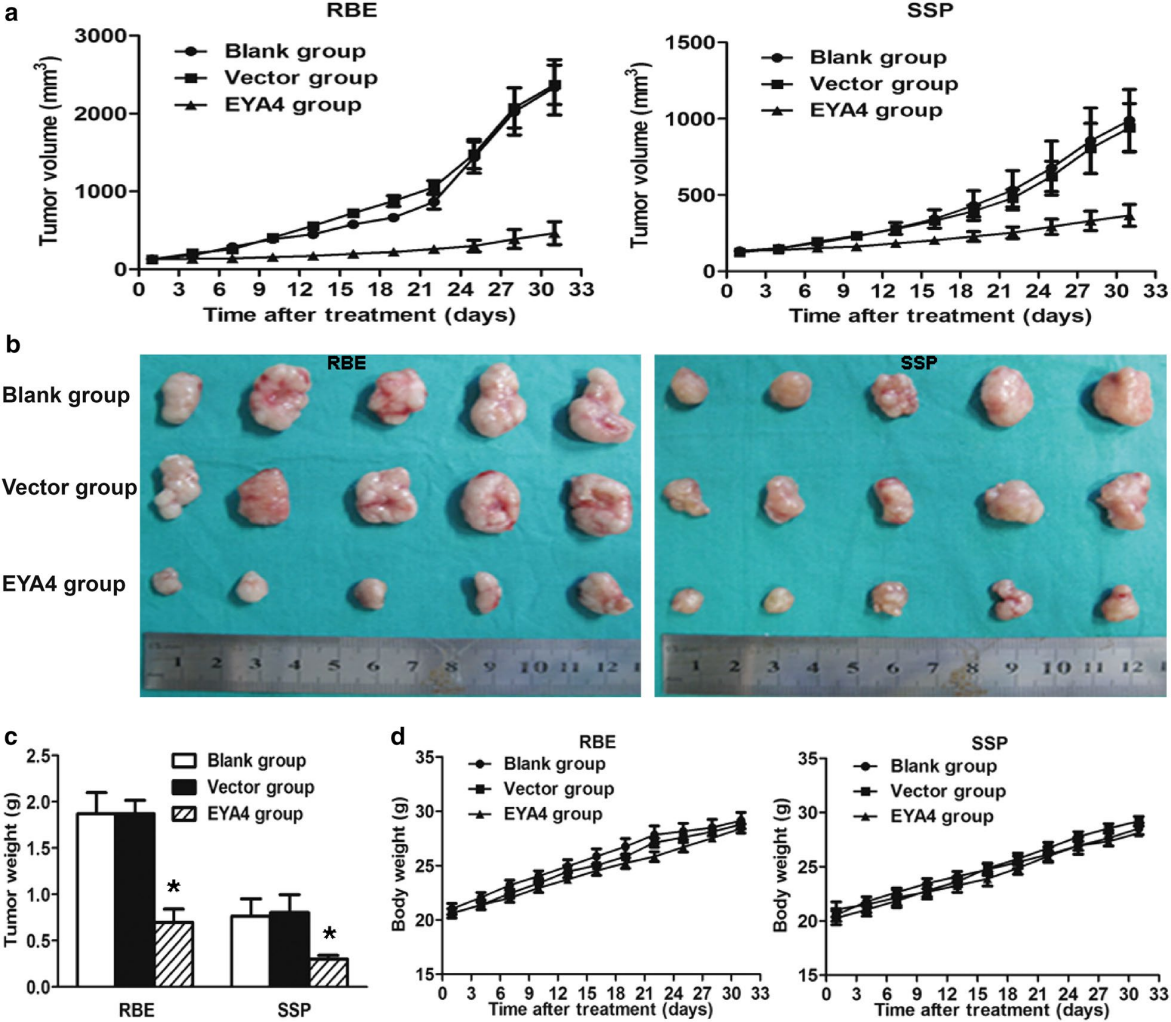
Intratumoral injection of *EYA4*-*expressing* plasmids suppressed RBE- and SSP-25-derived xenograft tumor growth in vivo. **a** Intra-tumoral injection of *EYA4* plasmids (EYA4 group) led to slower tumor growth at nearly all examined time points compared with vector and blank groups (*P* = 0.001 and *P* = 0.033, respectively); **b** After six treatments by intratumoral injection, the volume of tumors in the EYA4 group (*bottom*) was significantly smaller than those in the vector (*middle*) and blank groups (*top*, *P* = 0.001 for RBE and *P* = 0.011 for SSP, respectively). **c** Tumor weights in the EYA4 group were significantly smaller than those in vector and blank groups (*P = 0.004 for RBE and *P = 0.036 for SSP, respectively). **d** Adverse effects of *EYA4*-expressing plasmid treatment were evaluated by mice body weight, and no differences are observed among EYA4, vector and blank groups (*P* = 0.617 for RBE and *P* = 0.924 for SSP, respectively)

Intra-tumoral injection of *EYA4*-expressing plasmids (EYA4 group) significantly suppressed the growth of both RBE and SSP-25 xenograft ICC in NOD/SCID mice since day 3 after treatment as compared with vector plasmids (vector group) and blank control (blank group) (*P* = 0.001 for RBE and *P* = 0.033 for SSP-25; Fig. 4a). On day 30 after treatment, the mean volume of tumors in the EYA4 group was significantly smaller than those in the vector group and blank group, with a tumor volume of 463.8 ± 327.4 mm^3^ vs 2369.3 ± 564.3 mm^3^ and 2337.0 ± 693.6 mm^3^ in RBE xenograft tumors (*P* = 0.001; Fig. 4b), and 366.0 ± 161.2 mm^3^ vs 940.8 ± 355.9 mm^3^ and 988.8 ± 452.8 mm^3^ in SSP xenograft tumors (*P* = 0.011; Fig. 4b). Consistently, the weight of tumors in the EYA4 group was significantly lower than those in the vector and blank groups, both in RBE and SSP-25 xenograft tumors (*P* = 0.004 for RBE and *P* = 0.036 for SSP-25; Fig. 4c). With respect to the adverse effects of intra-tumoral injection of *EYA4*-expressing plasmids, there were no differences in the weights of mice among the three groups in both RBE tumors (*P* = 0.617) and SSP-25 tumors (*P* = 0.924; Fig. 4d). The in vivo results confirmed the tumor-suppressive effects of the *EYA4* gene on ICC cells.

## Discussion

In the present study, we showed that the expression of *EYA4* gene was down-regulated in ICC tissues and its expression level was an independent prognostic factor for patients with ICC undergone hepatectomy. Overexpression of *EYA4* gene could inhibit the growth of ICC cells in vitro and vivo. These results implied that the *EYA4* gene functioned as a molecular prognostic marker in ICC.

Recently, the *EYA4* gene was found to be hypermethylated in some malignancies, including esophageal, colorectal, and lung carcinoma [18–21]. In our previous study, we showed that the *EYA4* gene was hypermethylated with a down-regulated expression in HCC, and that low *EYA4* expression was an independent unfavorable prognostic factor [15]. In the present study, we showed that *EYA4* expression was notably lower in ICC tissues than in adjacent non-tumorous tissues. Additionally, EYA4 expression in ICC with multiple tumors or with lymph node metastases was significantly lower than that in ICC with single tumors or without lymph node metastases, respectively. Multivariate analysis showed that low EYA4 expression was an independent unfavorable prognostic factor for DFS and OS. These results suggest that the *EYA4* gene might be related to ICC tumorigenesis and progression and function as a molecular prognostic marker.

To investigate the biological functions of the *EYA4* gene on ICC, two stable *EYA4*-overexpressing transfectants (RBE-EYA4 and SSP-EYA4) were established. Compared with their corresponding vector controls, both RBE-EYA4 and SSP-EYA4 had significantly lower proliferation, foci forming, and invasiveness abilities. These results showed that overexpression of the *EYA4* gene could suppress growth of ICC cells in vitro. To further evaluate the biological effects of the *EYA4* gene on ICC in vivo, intra-tumoral injections of *EYA4*-expressing plasmids were administered to treat xenograft RBE and SSP-25 tumors in NOD/SCID mice. Compared with vector plasmid and blank control groups, intra-tumoral injection of *EYA4*-expressing plasmids significantly inhibited the growth of both xenograft RBE and SSP-25 tumors in NOD/SCID mice. These results showed that overexpression of the *EYA4* gene could suppress the growth of ICC. Additionally, it suggested that *EYA4* might function as a tumor suppressor gene in ICC. Our results were consistent with those reported by Kim et al. [20]. They found that *EYA4* overexpression could inhibit the growth of colorectal cancer cells both in vitro and in vivo. Using genome-wide expression array, they screened out abnormal expression of genes involved in the *Wnt*, mitogen-activated protein kinase (MAPK), and local adhesion signal pathways in *EYA4*-overexpressing colorectal cancer cells. Among these genes, they found that dickkopf WNT signaling pathway inhibitor 1 (*DKK1)*, an important inhibitor of the Wnt pathway, was significantly overexpressed in *EYA4*-overexpressing colorectal cancer cells; therefore, they speculated that *EYA4* could act as a tumor suppressor gene and inhibit the Wnt signal pathway in colorectal cancer cells by up-regulating *DKK1* [20]. However, further investigation is required to elucidate the mechanisms of *EYA4* in suppressing ICC.

In summary, we showed that the *EYA4* gene was a novel molecular prognostic marker for ICC and could inhibit the growth of ICC cells in vitro and vivo. However, the mechanism of the *EYA4* gene in inhibiting ICC cell growth remains unclear, and additional studies are needed.
